# Thermogenic Capacity of Human Supraclavicular Brown Fat and Cold-Stimulated Brain Glucose Metabolism

**DOI:** 10.3390/metabo13030387

**Published:** 2023-03-05

**Authors:** Mueez U-Din, Eleni Rebelos, Teemu Saari, Tarja Niemi, Katharina Kuellmer, Olli Eskola, Tobias Fromme, Johan Rajander, Markku Taittonen, Martin Klingenspor, Pirjo Nuutila, Lauri Nummenmaa, Kirsi A. Virtanen

**Affiliations:** 1 Turku PET Centre, Turku University Hospital, 20520 Turku, Finland; 2 Turku PET Centre, University of Turku, 20520 Turku, Finland; 3 Department of Plastic and General Surgery, Turku University Hospital, 20520 Turku, Finland; 4 Chair for Molecular Nutritional Medicine, Technical University Munich, 85354 Freising, Germany; 5 EKFZ—Else Kröner Fresenius Center for Nutritional Medicine, Technical University of Munich, 85354 Munich, Germany; 6 ZIEL—Institute for Food & Health, Technical University of Munich, 85354 Munich, Germany; 7 Radiopharmaceutical Chemistry Laboratory, Turku PET Centre, University of Turku, 20520 Turku, Finland; 8 Accelerator Laboratory, Turku PET Centre, Åbo Akademi University, 20520 Turku, Finland; 9 Department of Anesthesiology, Turku University Hospital, 20520 Turku, Finland; 10 Department of Endocrinology, Turku University Hospital, 20520 Turku, Finland

**Keywords:** human brown adipose tissue, BAT, cold stimulation, brain metabolism, thermogenesis, UCP-1

## Abstract

Human brain metabolism is susceptible to temperature changes. It has been suggested that the supraclavicular brown adipose tissue (BAT) protects the brain from these fluctuations by regulating heat production through the presence of uncoupling protein 1 (UCP-1). It remains unsolved whether inter-individual variation in the expression of *UCP-1*, which represents the thermogenic capacity of the supraclavicular BAT, is linked with brain metabolism during cold stress. Ten healthy human participants underwent ^18^F-FDG PET scanning of the brain under cold stimulus to determine brain glucose uptake (BGU). On a separate day, an excision biopsy of the supraclavicular fat—the fat proximal to the carotid arteries supplying the brain with warm blood—was performed to determine the mRNA expression of the thermogenic protein *UCP-1*. Expression of *UCP-1* in supraclavicular BAT was directly related to the whole brain glucose uptake rate determined under cold stimulation (*rho* = 0.71, *p* = 0.03). In sub-compartmental brain analysis, *UCP-1* expression in supraclavicular BAT was directly related to cold-stimulated glucose uptake rates in the hypothalamus, medulla, midbrain, limbic system, frontal lobe, occipital lobe, and parietal lobe (all *rho* ≥ 0.67, *p* < 0.05). These relationships were independent of body mass index and age. When analysing gene expressions of BAT secretome, we found a positive correlation between cold-stimulated BGU and *DIO2*. These findings provide evidence of functional links between brain metabolism under cold stimulation and *UCP-1* and *DIO2* expressions in BAT in humans. More research is needed to evaluate the importance of these findings in clinical outcomes, for instance, in examining the supporting role of BAT in cognitive functions under cold stress.

## 1. Introduction

The maintenance of bodily temperatures is necessary for the optimal performance of systemic metabolic processes for the endothermic animal species. The brain, being the master regulator of all bodily functions, also requires an optimal working temperature, and the metabolism of the brain changes linearly with the changes in intracranial temperatures, with a 6–8% change in every 1-degree Celsius change [1,2]. The neural activity also fluctuates with the changing intracranial temperatures in numerous mammals, including cats, monkeys, and rats [3,4,5].

Brain temperature is dependent on both the heat loss and the local heat production and the temperature of the arterial blood supplied to it, while the latter is found to be the major determinant in endothermic animals [6,7]. The local heat production in the brain depends on the rate of substrate utilisation and oxygen consumption, while the blood is supplied via the internal carotid arteries and vertebral arteries, which both arise from common carotid arteries. The location of these arteries, supplying the blood to the brain, is in proximity to the brown adipose tissue (BAT) depots in the neck and supraclavicular region. BAT, predominantly a thermogenic organ, plays a role in regulating body temperature, as evident from the fact that it is hypermetabolic under cold stress [8,9]. The supraclavicular location of BAT has been speculated to be of functional relevance for the brain, but so far, no data exist that may corroborate this hypothesis. BAT produces heat due to the presence of a unique protein, uncoupling protein-1 (UCP-1), present in the inner mitochondrial membrane. The extent of expression of this protein in BAT likely represents its heat-generating capacity; since it has been seen that UCP-1 is the only protein able to translocate protons via the inner membrane of mitochondria [10]. BAT metabolism is heterogenous in various populations [11], and previously, we have found that the metabolism of BAT is blunted in obesity [12,13]. Thus, it is unknown how inter-individual variation in BAT metabolic capacity may influence brain metabolism, particularly during hypothermic situations. Based on this, it is reasonable to hypothesise that BAT thermogenic capacity, as determined via UCP-1, is linked to brain metabolism under mild cold stress in healthy humans.

We have previously shown that the supraclavicular BAT glucose uptake (GU) rate is directly related to the brain glucose uptake (BGU) rate during cold stress [14], suggesting that the brain plays a role in regulating BAT metabolism. Given BAT’s possible role in endocrine signalling [15], the association of BAT’s heat-generating capacity or its secretome to the brain metabolism have not been evaluated. Therefore, in this study, as an extension of previous work, we examined the mRNA expression of the heat-producing protein UCP-1 in BAT, as well as those linked to BAT secretome profile [15] and investigated their links with cold-stimulated brain glucose metabolism.

## 2. Materials and Methods

### 2.1. Human Study Participants and Study Design

Ten volunteers were recruited from Turku, Finland, via electronic and newspaper advertisements. All subjects were healthy as determined by laboratory tests, a 2-h oral glucose tolerance test, electrocardiography, blood pressure measurements and medical history. A hyperinsulinemic-euglycemic clamp technique was also used to measure whole-body insulin sensitivity (M-value). The anthropometric characteristics of the study participants have been given in Table 1. Written informed consent was obtained from all subjects prior to inclusion. The study protocol was approved by the Ethics committee of the hospital district of Southwest Finland. The study was carried out according to the principles of the Declaration of Helsinki and GCP guidelines.

### 2.2. Brain PET Imaging and Analysis

**Figure 1:**
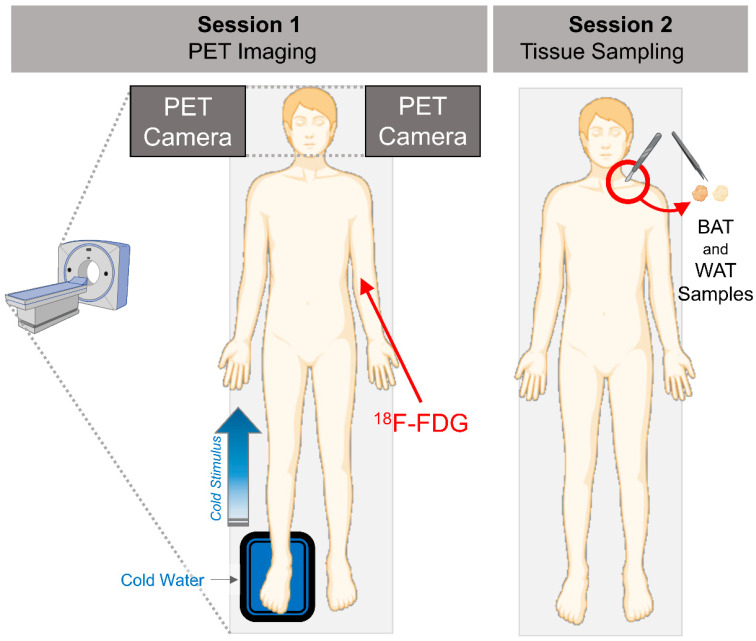
In session 1, PET scanning using an ^18^F-FDG radiotracer was conducted with cold water foot immersion to give cold stimulus to the participants. In session 2, an excision biopsy of the fat from the supraclavicular neck region was obtained.

Positron emission tomography (PET) was performed under fasting conditions. Before PET imaging, study participants spent 2 h in a room with an ambient temperature of 17 °C. During the imaging session, the participants were placed supine in the PET-CT scanner and the right foot of the participants was immersed in cold water (8 °C) in intervals of 5 min (5 min in water and 5 min out of water—Figure 1). Dynamic brain PET-image acquisition (5 × 3 min frames) took place ~90 min after a bolus injection of (^18^F) 2-fluoro-2-deoxy-D-glucose (^18^F-FDG). For emission data acquisition, GE Discovery VCT (General Electric Medical Systems, Milwaukee, WI, USA) and ECAT EXACT HR+ scanners (Siemens/CTI, Knoxville, TN, USA) were used. The PET data were corrected for photon attenuation, physical decay, dead time, scatter, and random coincidences, and PET images were reconstructed with a matrix size of 128 × 128 using iterative reconstructions.

Voxel-based mapping of BGU was performed with SPM8 (http://www.fil.ion.ucl.ac.uk/spm/). Parametric BGU images were calculated voxel by voxel. The dynamic brain PET images were summed and normalized into MNI standard space; subsequently, these parameters were applied to the parametric BGU images. Spatially normalized BGU images were smoothed with a 12-mm full width at a half-maximum Gaussian kernel. Linear regressions were performed in SPM to evaluate correlations between BGU and single regressors (BAT *UCP-1* expression, BAT secretomes gene expression, and BAT-probability score). A statistical threshold of *p* < 0.05 with false discovery rate correction at cluster levels was applied in all analyses. BGU values were also generated and extracted for the following regions of interest: frontal cortex, parietal cortex, temporal cortex, occipital cortex, limbic lobe, pons, sub lobar regions, hypothalamus, and cerebellum using the WFU Pickatlas tool (Wake Forest University Health Sciences Center, Winston-Salem, NC 27157, USA).

### 2.3. Adipose Tissue Biopsies

BAT and WAT tissue samples were collected from participants under local lidocaine-epinephrine anaesthesia. The site of the supraclavicular BAT biopsy was determined from imaging data, and subcutaneous WAT was obtained from the same opening. An experienced plastic surgeon carried out this procedure. Immediately after being collected, the samples were snap-frozen in liquid nitrogen. The tissue samples were acquired at room temperature. RNA isolation and next-generation sequencing were performed as previously described [16].

### 2.4. Statistical Analyses

Statistical analyses were performed using IBM SPSS statistics (version 28.0.1.0), and the graphical plots and heatmaps were created using Graphpad Prism (version 6.01) and R-studio (version 1.2.5042), respectively. For paired sample comparison (gene expression in BAT vs. WAT), a paired *t*-test was used for normally distributed datasets, and where datasets did not show a normal distribution, a Wilcoxon rank-sum test was used. In correlation analyses, Spearman’s correlation test was used. Brain analysis at the voxel and cluster level was performed with statistical parametric mapping (SPM) running on Matlab for windows (version 9.1.0; Math Works, Natick, MA, USA). A *p*-value of <0.05 was considered significant.

## 3. Results

### 3.1. Supraclavicular BAT UCP-1 Expression and Cold-Stimulated Brain Glucose Uptake

**Figure 2:**
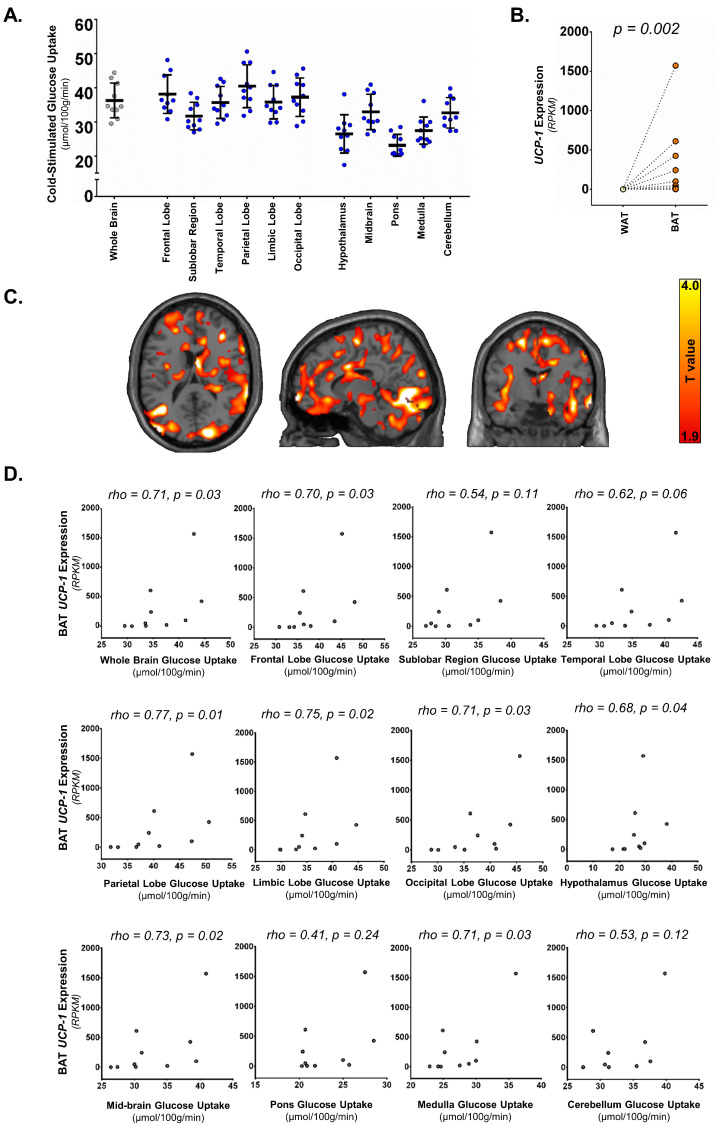
(**A**) Brain glucose uptake rate after a mild cold stimulus determined using ^18^F-FDG PET imaging. (**B**) The expression of the *UCP-1* gene in supraclavicular BAT and subcutaneous neck WAT. (**C**) Statistical parametric mapping (SPM) images of the association between brain glucose uptake (BGU) during cold conditions and BAT *UCP-1* expression. *p* values < 0.05 cluster level; *p* values < 0.05 voxel level; extend threshold k = 114 voxels; FDR-corrected. Higher T values denote stronger associations (coloured bar at the side). (**D**) The relationship between *UCP-1* expression in supraclavicular BAT and brain glucose uptake in the whole brain and different brain regions.

The whole-brain GU rate under cold stress ranged from 29.6 to 44.3 µmol 100 g^−^^1^ min^−^^1^ (mean ± SD: 36.3 ± 5.1 µmol 100 g^−^^1^ min^−^^1^, median: 34.6 µmol 100 g^−^^1^ min^−^^1^), showing a certain degree of inter-individual variability in a healthy population. The GU in the whole brain and individual brain compartments have been shown in Figure 2A. The whole brain GU, as well as GU in sub-compartments in the brain, were not related to BMI or age in Spearman’s correlation analysis (BMI: *rho* = −0.38, *p* = 0.33; age: *rho* = −0.21, *p* = 0.56).

The gene expression of *UCP-1* was significantly higher in the supraclavicular fat depot (BAT) compared to neck subcutaneous white adipose tissue (WAT), confirming the presence of thermogenic adipose tissue in the supraclavicular depot (Figure 2B). The expression of the *UCP-1* gene in the supraclavicular region was found to be directly related to whole-brain GU (Figure 2C,D), as well as to the cold stimulated GU of the frontal lobe, parietal lobe, limbic lobe, occipital lobe, hypothalamus, midbrain, and medulla (Figure 2D). There was a tendency toward significance for the cold-stimulated temporal lobe GU (Figure 2D). There was no significant relationship with cold-stimulated GU of the sub lobar region, pons, and cerebellum (Figure 2D). The relationships between BGU and BAT expression of *UCP-1* remained significant once adjusted for either BMI or age in nonparametric partial correlation analysis (Table 2). There was no relationship between the expression of *UCP-1* in subcutaneous WAT and BGU.

### 3.2. Supraclavicular BAT Secretome Genes, BAT-Probability Score and Brain Glucose Uptake

**Figure 3:**
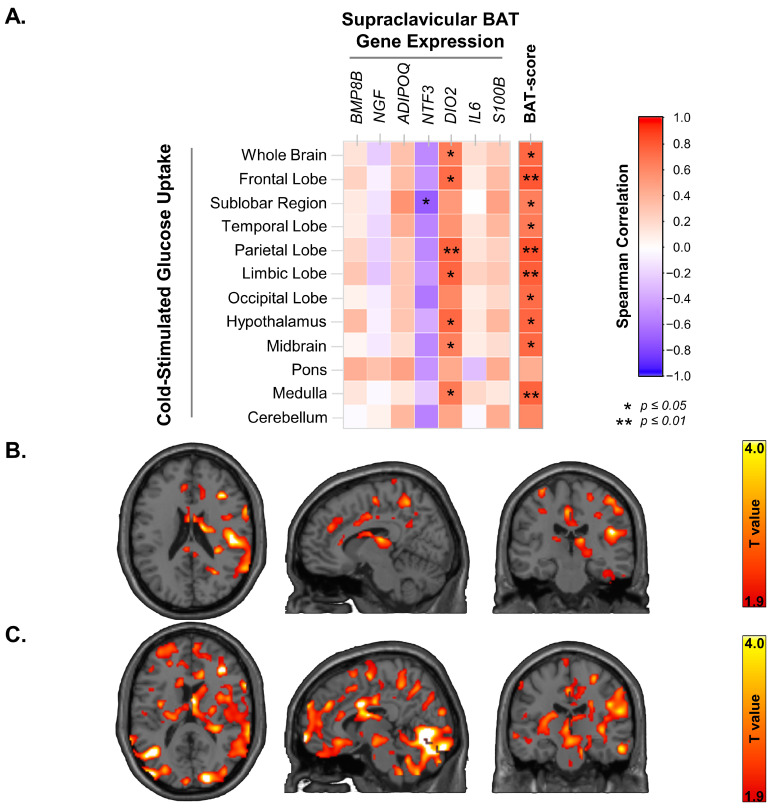
(**A**) The relationship between the expressions of genes in supraclavicular BAT—that may influence brain metabolism via proteins encoded by them—and brain glucose uptake in mild cold stress. The relationship between BAT-probability score—based on the transcriptional profile of supraclavicular BAT sample, and cold stimulated brain glucose uptake. (**B**) Statistical parametric mapping (SPM) images of the association between brain glucose uptake (BGU) during cold conditions and BAT *DIO2* expression. *p* values < 0.05 cluster level; *p* values < 0.05 voxel level; extend threshold k = 3031 voxels; FDR-corrected. Higher T values denote stronger associations (coloured bar at the side) (**C**) Statistical parametric mapping (SPM) images of the association between brain glucose uptake (BGU) during cold conditions and BAT-probability score. *p* values < 0.05 cluster level; *p* values < 0.05 voxel level; extend threshold k = 114 voxels FDR-corrected. Higher T values denote stronger associations (coloured bar at the side).

BAT has been suggested to have endocrine effects on several tissues via secretory factors; we investigated the expression of genes that may hold relevance to brain metabolism and investigated their relationship to cold-stimulated brain glucose metabolism. We examined the expressions of the genes encoding Bone Morphogenetic Protein 8b (BMP8B), Nerve growth factor (NGF), Neurotrophin-3 (NTF3), Type II iodothyronine deiodinase (DIO2), Interleukin 6 (IL-6) and S100 calcium-binding protein B (S100B) in both supraclavicular BAT and subcutaneous neck WAT. Further, we also calculated the BAT-probability score using the ProFAT analysis tool [17] based on the transcriptional profile of the BAT samples from the supraclavicular region. The expression of *BMP8B* and *DIO2* was significantly higher in BAT as compared to WAT, while no significant differences were observed in the expression of *NRG4*, *S100B*, *NGF*, *NTF3* and *IL6*. The expression of *DIO2* was directly related to the whole brain GU (*rho* = 0.66, *p* = 0.038), as well as to the GU of several sub-compartments of the brain, including the hypothalamus, medulla, midbrain, limbic lobe, frontal lobe, and parietal lobe (Figure 3A,B). There were no significant relationships between the expression of these genes in WAT and BGU. BAT-probability score was also significantly related to whole-BGU (Figure 3A,C) and to the GU of several sub-compartments of the brain, including the hypothalamus, medulla, midbrain, sub lobar, frontal lobe, limbic lobe, occipital lobe, temporal lobe and parietal lobe (Figure 3A).

## 4. Discussion

The main findings of the present study are that BGU during cold stress correlates with the expression of *UCP-1* in supraclavicular BAT, showing a physiological connection between the brain and BAT. Moreover, when assessing the BAT secretome genes, we found that the expression of the gene encoding iodothyronine deiodinase 2 (*DIO2*) was also associated with BGU. Also, the BAT-probability score, based on the transcriptional profile of excised BAT from the supraclavicular region, was found to be strongly linked to cold-stimulated BGU. These results require elucidation in the context of previous findings on this topic.

In our previous publication by Orava et al. (2014), we have shown that cold stimulation increases BGU, and it correlates positively with cold-stimulated BAT GU [14]. Thus, in the present investigation, we pursued to assess whether BAT’s thermogenic capacity, as indicated by the expression of *UCP-1*, is also associated with BGU. Indeed, we found a direct relationship between cold-stimulated BGU and *UCP-1*, suggesting that not only are the two tissues metabolically linked during cold stress [14] but also that brain metabolism under mild cold stress is linked with BAT capacity to generate heat in healthy adults. The brain areas significantly associated with *UCP-1* expression of BAT are shown in Figure 2C and are listed in Table 2, including the frontal, parietal, limbic, and occipital lobes, as well as the hypothalamus, midbrain, and medulla.

The brain operates at a slightly warmer temperature than the rest of the body; Soukup et al. (2004) show that the temperature of deeper brain regions, on average, is approximately 1 °C higher than the rest of the body [18]. Physiological fluctuations in the temperature range of 2–4 °C have been observed in the brains of various endotherms [4,19]. Variations in brain temperature affect the metabolic rates of the brain through multiple mechanisms [20]. As the brain temperature is predominantly dependent on the temperature of the carotid blood supply [7], the functional importance of BAT in providing the brain with warm blood has been long speculated. Our results showing a direct link between BAT thermogenic capacity and BGU in cold conditions could be taken as evidence of the critical role of BAT in this physiological regulation.

The hypothalamus, specifically the preoptic anterior hypothalamus, is considered the coordinating integration centre for thermoregulation [21,22,23]. Although quantifying hypothalamic metabolism with PET is difficult [24], our data suggest a significant role of the hypothalamus in the BAT-brain connection but also reveal that several other brain regions are involved as well. Indeed, important nodes of the thermoregulation network are also considered the periaqueductal grey in the midbrain and the nucleus raphe pallidus in the medulla [25], with midbrain and medulla GU rates in the present study being significantly associated with BAT *UCP-1* expression. Whether cortical regions also participate in thermoregulation is less clear. However, since ^18^F-FDG-PET indexes brain substrate metabolism rather than neuronal firings, the positive association between BAT *UCP-1* expression and cortical GU rates may indicate either the participation of cortical regions in the thermoregulatory control or that the metabolic rates in the cortical region, as in other regions, are linked to the extent of heat generating ability of BAT during cold stimulation.

**Figure 4:**
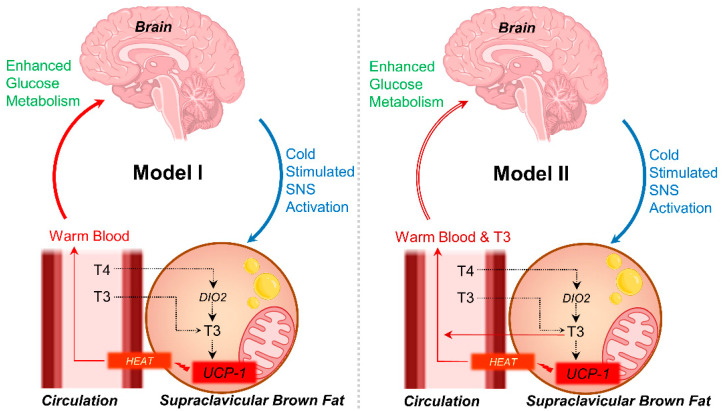
Speculated physiological models (I and II) based on data shown in the current report that explain how BAT may influence brain metabolism.

The relationship between the expression of *DIO2* in BAT and cold-stimulated BGU is also of significant interest. DIO2 catalyses the transformation of the pre-hormone thyroxine (T4) to 3,5,3′-triiodothyronine (T3). In humans, DIO2 is expressed in the brain, BAT, uterus, placenta, thyroid gland, and heart. Under normal conditions, the primary circulating thyroid hormone is T4, and DIO2 contributes largely to circulating T3 and fulfills the intracellular needs of thyroid hormones. Considering the location of BAT is in close proximity to the brain, and the finding of a positive link between BAT expression of *DIO2* and BGU, it is tempting to speculate that the increased expression of DIO2 in BAT may serve to provide T3 to the brain, along with the warm blood (Figure 4). This mechanism could be essential for supporting the T3 needs of the brain during cold stress [26] via circulation [27,28] or providing thermogenic feedback communication to the brain [29]. Previous studies have shown that T3 mediates glucose metabolism in brain astrocytes, as the primary cultures of rodents devoid of L-T3 have low rates of 2-deoxyglucose uptake [30], and when astrocytes are replenished with L-T3 the 2-deoxyglucose uptake is enhanced, increasing the binding sites of the glucose transporter [31]. As ^18^F-FDG uptake in the brain is mainly driven by astrocytes rather than neurons [32], it is possible that systemic DIO2 expression plays a role in regulating BGU, particularly in the challenging situation of mitigating hypothermia. However, it cannot be ruled out that T3 generated by BAT could be merely to support UCP-1 activation [33,34,35], or it may also act as a communication feedback loop between BAT and the brain to regulate thermogenesis. In the same context, López et al. (2010) have noticed that central administration of T3 in rats leads to the activation of SNS and BAT. Also, in humans, we have noticed that hyperthyroidism leads to upregulated BAT metabolism [36]. The plausibility of the co-occurrence of these mechanisms also exists.

We also found a link between BAT gene expressions, BAT-probability score, and cold-stimulated glucose uptake of the brain, further strengthening the hypothesis of a metabolic link from the supraclavicular BAT to the brain. Previously, our institute, in collaboration with Klingenspor’s laboratory, has concretely shown that the gut hormone secretin activates BAT and induces satiation via BAT-to-Brain communication [37,38]. This communication between the BAT and brain to induce satiation likely happens through BAT’s heat generation in the postprandial state [16]. Our data suggest that BAT-to-brain communication may also occur during other physiological conditions, i.e., cold. These findings support the hypothesized functional BAT-brain axis, where BAT may communicate to the brain via heat production or via secreted factors. In this context, BAT may exert a positive influence to support glucose metabolism in the brain during cold stress, in line with our previous observations that BAT protects the brain from cognitive degeneration associated with cardiometabolic risk [39].

Our results have further implications, where data suggests that brain cognitive performance is adversely affected by frequently changing air temperatures [40], implying that the ability to maintain a stable brain temperature may have a link to cognitive performance. In a similar standpoint, and in the context of our findings, it can be speculated that healthy human adults with the capacity to actively regulate brain temperatures via active BAT depots may have better cognitive ability in cold environments. These findings may find applications in soldiers, divers, fighter jet pilots, astronauts, and other professionals who need to make intelligent decisions in fluctuating cold weather environments. The recruitment and activation of brown adipose tissue via BAT-selective pharmacological agents can be a meaningful approach for enhancing cognitive performance under cold stress; future studies are needed to systematically test this hypothesis.

The novelty of this study consists of the combination of human brain imaging and human BAT excision biopsy measurements to correlate brain metabolism with *UCP-1* expression and BAT’s secretome genes. Nevertheless, our study is not without limitations. The study is cross-sectional; hence, the causal links between BAT thermogenic capacity and BGU cannot be determined. Additionally, due to ethical constraints, the tissue excision from the supraclavicular fat depot was done without exposing the study participants to cold, limiting us from assessing the expression of genes under cold stress. Further, due to a lack of technological advancement in the robust measurements of intracranial temperatures, it remains unclear in humans whether glucose metabolism in the brain is associated with intracranial temperatures. Future studies in humans should focus on measuring intracranial brain temperature and brain substrate metabolism simultaneously to fully understand how variations in brain temperature affect the uptake and metabolism of different nutrients. However, the results shown here align with other published literature, and they advance our current understanding of the role BAT thermogenic function has in aiding brain metabolism in adult humans. Finally, hypothalamic GU values should be considered with caution since the assessment of tiny brain regions is hampered by the spatial resolution of the PET (6–8 mm) [24].

## 5. Conclusions

Altogether, our results reinforce the hypothesis that supraclavicular BAT in adult humans provides physiological assistance to the brain metabolism during cold stress. The support of the BAT to the brain metabolism is via its heat-generating properties and likely also through its secretome. Future studies are required that may elucidate whether or not adult humans with a substantial amount of BAT have a superior cognitive performance during cold stress.

## Figures and Tables

**Table 1:** Anthropometric Characteristics of Study Participants.

N	10
Age, years	39.2 ± 8.3
Male/Female, n	1/9
BMI, kg/m^2^	24.4 ± 4.5
Waist, cm	81.4 ± 19.1
Fasting Plasma TSH, mU/L	2.4 ± 0.8
Fasting Plasma T4, pmol/L	15.0 ± 2.5
Fasting Plasma T3, pmol/L	4.9 ± 0.7
Insulin sensitivity (M-value) (μmol/kg/min)	46.3 ± 27.6

Data are presented as mean ± SD.

**Table 2:** Partial Correlations Analysis BAT *UCP-1* expression and Cold-Stimulated Brain Glucose Uptake Rate.

Glucose Uptake Rates	Correlation Adjusted for BMI		Correlation Adjusted for Age	
	*rho*	*p*-Value	*rho*	*p*-Value
Whole Brain	**0.72**	**0.029**	**0.71**	**0.03**
Frontal Lobe	**0.70**	**0.034**	**0.70**	**0.04**
Sublobar Region	0.54	0.13	0.54	0.13
Temporal Lobe	0.63	0.07	**0.62**	**0.07**
Parietal Lobe	**0.78**	**0.012**	**0.77**	**0.016**
Limbic Lobe	**0.73**	**0.026**	**0.72**	**0.029**
Occipital Lobe	**0.73**	**0.026**	**0.73**	**0.026**
Hypothalamus	**0.68**	**0.046**	**0.67**	**0.048**
Midbrain	**0.73**	**0.025**	**0.76**	**0.018**
Pons	0.41	0.27	0.44	0.23
Medulla	**0.71**	**0.03**	**0.72**	**0.03**
Cerebellum	0.53	0.14	0.55	0.12

## Data Availability

Data are available from the corresponding author, after reasonable request. Data is not publicly available due to privacy or ethical restrictions.
